# Cortical AAV-CNTF Gene Therapy Combined with Intraspinal Mesenchymal Precursor Cell Transplantation Promotes Functional and Morphological Outcomes after Spinal Cord Injury in Adult Rats

**DOI:** 10.1155/2018/9828725

**Published:** 2018-08-06

**Authors:** Stuart I. Hodgetts, Jun Han Yoon, Alysia Fogliani, Emmanuel A. Akinpelu, Danii Baron-Heeris, Imke G. J. Houwers, Lachlan P. G. Wheeler, Bernadette T. Majda, Sreya Santhakumar, Sarah J. Lovett, Emma Duce, Margaret A. Pollett, Tylie M. Wiseman, Brooke Fehily, Alan R. Harvey

**Affiliations:** ^1^School of Human Sciences, The University of Western Australia (UWA), Perth, WA 6009, Australia; ^2^Perron Institute for Neurological and Translational Science, Nedlands, WA 6009, Australia; ^3^University of Notre Dame Australia, Fremantle, WA 6959, Australia

## Abstract

Ciliary neurotrophic factor (CNTF) promotes survival and enhances long-distance regeneration of injured axons in parts of the adult CNS. Here we tested whether CNTF gene therapy targeting corticospinal neurons (CSN) in motor-related regions of the cerebral cortex promotes plasticity and regrowth of axons projecting into the female adult F344 rat spinal cord after moderate thoracic (T10) contusion injury (SCI). Cortical neurons were transduced with a bi*cis*tronic adeno-associated viral vector (AAV1) expressing a secretory form of CNTF coupled to mCHERRY (AAV-CNTF^mCherry^) or with control AAV only (AAV-GFP) two weeks prior to SCI. In some animals, viable or nonviable F344 rat mesenchymal precursor cells (rMPCs) were injected into the lesion site two weeks after SCI to modulate the inhibitory environment. Treatment with AAV-CNTF^mCherry^, as well as with AAV-CNTF^mCherry^ combined with rMPCs, yielded functional improvements over AAV-GFP alone, as assessed by open-field and Ladderwalk analyses. Cyst size was significantly reduced in the AAV-CNTF^mCherry^ plus viable rMPC treatment group. Cortical injections of biotinylated dextran amine (BDA) revealed more BDA-stained axons rostral and alongside cysts in the AAV-CNTF^mCherry^ versus AAV-GFP groups. After AAV-CNTF^mCherry^ treatments, many sprouting mCherry-immunopositive axons were seen rostral to the SCI, and axons were also occasionally found caudal to the injury site. These data suggest that CNTF has the potential to enhance corticospinal repair by transducing parent CNS populations.

## 1. Introduction

Most spinal cord injury (SCI) results from contusion rather than transection injuries, and cervical injuries (~60–70% of all SCI) produce greater deficits and threaten more critical survival systems than thoracic/lumbar SCI. The corticospinal tract (CST) is important in the control of voluntary skilled movements, especially of distal limbs. Human CST projections are not completely homologous to the descending CST in rodents (which in these species projects mainly to the forelimbs and is mainly located in the dorsal rather than lateral columns) [1], nonetheless, because of the importance of CST projections in fine manipulatory motor control, this pathway has been a focus of many experimental repair strategies aimed at restoring function following SCI. Most studies in rodents have attempted this by the delivery of purified neurotrophic growth factors and/or by cell transplantation using donor cells engineered to overexpress the growth factors, the factors usually applied to the injury site itself (see [2]). In such studies, functional improvements usually reflect sprouting and some plasticity in collateral and/or intraspinal pathways ([3, 4], c.f. [5]), rather than axonal regeneration per se.

Our gene therapy approach targets corticospinal neurons (CSN) and is aimed at enhancing axonal plasticity and inducing regeneration of CST axons, leading to behavioural improvements after SCI. Gene therapy involves the transduction of neurons in the sensorimotor cortex using a bi*cis*tronic adeno-associated viral vector (AAV) that encodes and expresses a secretory form of ciliary neurotrophic factor (CNTF) coupled to mCHERRY (AAV-CNTF^mCherry^). Injection of AAV-CNTF^mCherry^ into the cortical regions of the brain that project onto the output pathways of the CST allows expression of CNTF in neurons, including CSN, at the time of SCI.

CNTF has been selected because it is known to promote the survival of injured CSN [6], and in other systems it promotes long-distance regeneration of injured adult CNS axons [7–10], with at least some functional outcomes [11]. CNTF is a neuropoietic member of the interleukin 6 (IL-6) cytokine family, expressed primarily in glial cells of the nervous system [12–15]. Survival effects of CNTF have been demonstrated on motor, retinal ganglion, cortical and hippocampal, red nucleus, and striatal and thalamic neuron populations [14–16] (see also [17]). CNTF has demonstrated efficacy in limiting neuronal injury in several experimental disease and injury paradigms, and has been clinically evaluated as a treatment for motor neuron-specific amyotrophic lateral sclerosis (ALS) [18, 19]. CNTF protects corticospinal neurons in the sensorimotor cortex after intracortical axotomy [6], and these neurons, at least in murine neonates, are known to express CNTF receptor *α* [20]. Functional improvement and enhanced remyelination has also been reported after intraspinal transplantation of oligodendrocyte precursor cells expressing CNTF [21]. However, as we have argued [2], while most SCI studies have targeted the injured cord itself for therapy, in other systems—such as the visual system—targeting the injured neurons themselves yields excellent functional outcomes [11, 22]. Most importantly there is now clear evidence in human postmortem material that there is long-term survival of CSN after SCI [23], making strategies that target these neurons of genuine clinical relevance, potentially in both acute and chronic circumstances.

In an initial study, we obtained preliminary evidence that AAV2.1-CNTF^mCherry^ transduced large numbers of neurons in the sensorimotor cortex containing CSNs projecting into the spinal cord, and after moderate thoracic T10 contusion SCI there was clear and consistent sprouting of mCherry-labelled CST axons at, and rostral to, the lesion site [2]. In the present report, cortical gene therapy has been also combined with the transplantation of mesenchymal precursor cells (MPCs) into the injured thoracic cord, a method shown by us and others consistently to limit tissue loss and promote morphological sparing as well as functional improvement following SCI [24, 25]. The rationale is that a healthier local environment at the cell-transplant injury site provides a better terrain for plasticity and regeneration of CST axons after targeted CNTF expression in the motor cortex. MPC treatment may be especially needed for contusion injuries which tend to be more unstable and prone to cyst formation—this is vital when treating cervical injuries in humans due to ongoing cavity formation (syringomyelia) and consequent progressive, sometimes catastrophic, loss of function [26].

## 2. Materials and Methods

### 2.1. Animals and Experimental Design

Adult female Fischer (F344) rats (age 10–12 wk, 120–150 g; Animal Resource Centre, Western Australia) were used in experimental procedures conforming to National Health and Medical Research Council Guidelines (Australia) and approved by the University of Western Australia Animal Ethics Committee. A total of 43 rats was used, distributed between 4 experimental SCI groups as follows; SCI + control AAV-GFP (*n* = 11), SCI + AAV-CNTF^mCherry^ (*n* = 16), SCI + AAV-CNTF^mCherry^ + nonviable rMPCs (*n* = 6), and SCI + AAV-CNTF^mCherry^ + viable rMPCs (*n* = 11). Previous experiments conducted in our lab used SCI + either nonviable or viable rMPC cell transplantation only [24, 25], and to satisfy NHMRC guidelines to address animal welfare and minimise animal use, these groups were not repeated for the present study. Viral AAV transduction was performed 2 weeks prior to SCI, and 2 weeks prior to experimental endpoint, biotinylated dextran amine BDA conjugated to horseradish peroxidase (Thermo Fisher Scientific) was spaced and injected in virtually identical positions as the earlier AAV injections in order to cover the same hindlimb projection fields in the sensorimotor cortex. Endpoint was reached and animals culled at day 56 after SCI.

#### 2.1.1. Viral Vectors

The bi*cis*tronic adeno-associated viral vector (AAV) encoding and expressing a secretory form of CNTF coupled to mCHERRY (AAV-CNTF^mCherry^) or vector-alone control linked to green fluorescent protein (AAV-GFP) were made by Vector Biolabs, USA. AAV vectors consisted of an AAV2 DNA backbone in an AAV1 capsid. This particular serotype has been shown to provide excellent transduction of cortical neurons [27–29]. Transgene expression was driven by a shortened CAG2 promoter based on the typical cytomegalovirus/chicken beta-actin (CAG) promoter. For bicistronic vectors, the transgene (CNTF) and reporter (mCherry) were linked by a 2A viral peptide sequence, which causes a “translational skip” and results in a 1 : 1 expression of transgene and reporter proteins. The CNTF transgene is a mouse CNTF gene preceded by the secretory signal sequence from mouse pre-pro-NGF, to allow for local secretion of CNTF [30] (a gift from Prof. M. Sendtner, University of Wurzburg, Germany).

#### 2.1.2. Viral Transduction of Hindlimb Sensorimotor Cortex

AAV-CNTF^mCherry^ or AAV-GFP transduction of CSN in the cortical regions that project axons to the level of the spinal cord that controls the hindlimbs [2, 31] was performed 2 weeks prior to SCI via 4 × 0.5 *μ*l injections of the respective virus (~4 × 10^13^ genomic copies/ml) using a nanojet device and a digital stereotaxic frame (Kopf) for accurate Bregma/Lambda coordinate positioning. Injection of AAV 2 weeks prior to SCI allowed time for onset of transgene expression and production of CNTF.

### 2.2. Spinal Cord Injury (SCI)

Rats were anesthetized with 1.5% (*v*/*v*) halothane (Rhone-Poulenc Chemicals Pty. Ltd., Australia) combined with nitrous oxide (60%) and oxygen (38.5%). Amacin ophthalmic eye ointment was applied before rats were placed on a heating pad (37°C). Partial laminectomy at vertebral level T9-T10 exposed the SC underneath without disrupting the dura [24, 25]. Using an Infinite Horizon impactor device, a 200 kDyne contusion injury was induced at the exposed spinal cord. Postsurgery care, analgesics, food, and housing were as previously described [24, 25]. Briefly, rats were treated with Benacillin (0.02 ml/100 g body wt., 300 U/ml, i.m.) and painkiller Buprenorphine (Temgesic, 0.01 ml/100 g, 300 U/ml, ip) for 5 days.

### 2.3. Donor rMPCs and Transplantation

Commercially available rMPCs isolated from Fischer F344 rats (Cyagen Biosciences Inc., number RAFMX-01201) were routinely maintained in Mesenchymal Stem Cell Growth Medium (number GUXMX-90011) prior to use in transplantation experiments, or for those used for routine phenotypic characterization and differentiation [24, 25] maintained for at least 24–48 hr, in order to determine any neuronal phenotype/marker expression using the panel of antibodies described in Section 2.7.2. For transplantation experiments, MPCs (no higher than passage number 5) were washed and resuspended in PBS. Nonviable MPCs were prepared by multiple (×3) freeze/thawing steps between −80°C and 37°C and confirming loss of viability using trypan blue staining under microscopy. At day 14 after SCI, 6 × 10^5^ cells in a total of 4 *μ*l were injected in a single injection directly into the lesion site (rostrocaudally at 1 mm depth) through a finely drawn (80 *μ*m tip) glass pipette connected to a 10 *μ*l Hamilton syringe and driven by a Harvard Pump at 0.5 *μ*l/min (total duration is 8 min). The pipette was left in place to prevent cell leakage for 1 min before withdrawal [24, 25].

### 2.4. BDA Injection into Sensorimotor Cortex that Projects to Low Thoracic/Lumbar Spinal Cord

Injections of biotinylated dextran amine were spaced in virtually identical positions as the earlier AAV-CNTF^mCherry^ or AAV-GFP transduction injections in order to cover the same hindlimb projection fields in the sensorimotor cortex. For each of the 4 injections, 0.5–1 *μ*l of 10% (*w*/*v*) BDA were injected using a nanojet device (World Precision Instruments) at a depth of 1 mm. The location of these injections is based on studies showing the location of CMN that project to the hindlimbs and forelimbs in Fischer rats [2] and confirmed by the examination of the label in appropriate thalamic motor nuclei. In some rats, gelfoam soaked in the BDA solution was placed over the exposed cortex prior to the closure of the craniotomy.

#### 2.5. Functional Behaviour

A variety of behavioural tests for injured rats were used to give a valid indication of functional recovery [24, 25, 32–35] including (i) “open-field” locomotion test (BBB scoring method [36] to assess spontaneous movements), (ii) ladder walking [37] to assess general hindlimb recovery, and (iii) our own novel computerized quantitative gait analysis method (Ratwalk® [38]) which allows objective analyses of a large number of locomotion parameters, such as interlimb coordination (step sequence), stride length, step length, and base of support (stance width) [38]. All functional (locomotor) behaviour was assessed weekly for up to day 56 after SCI. BBB scoring was analysed using at least 3 blinded raters both at the time of assessment and later *in silica* via slow motion/frame-by-frame replay of high definition (1080 p) digital recordings taken during the test. The BBB rating score is composed of 22 nonlinear operational definitions (0–21 scale) studying several aspects involved in the locomotion of quadrupedal animals such as weight support, plantar stepping, and forelimb/hindlimb coordination. Ladderwalk and Ratwalk assays were performed on animals once they had reached weight support on their hindlimbs, and involved comparison of preinjury performance with weekly assessments from day 14 post-SCI until day 56 (endpoint). Digital recordings of animals traversing 3 lengths per time point of Ladderwalk and Ratwalk (preinjury and weekly up to day 56 post-SCI) were also similarly assessed by at least 2 blinded raters *in silica* via slow motion/frame-by-frame replay of digital recordings taken during the tests for preinjury and up to day 56 post-SCI. Ladderwalk scores are an average of missteps over a 1 m horizontal ladder with unevenly spaced bars. Ratwalk data analysis involves frame by frame designation from digital recordings of left and right fore- and hindlimb placement on a 1 m glass platform in the Ratwalk apparatus in low light, using an average of 3 “runs” per time point [38].

### 2.6. Perfusion and Tissue Processing

At day 56 (2 weeks after the BDA cortex injections), rats were euthanized by lethal injection of sodium pentobarbitone (50 mg/100 g) and transcardially perfused in 0.9 M with 100 ml of heparinized Dulbecco's PBS followed by 4% (*w*/*v*) paraformaldehyde in PBS pH 7.4. The head and vertebral column were dissected from each animal and postfixed for 24 hours. The brain and spinal cord were extracted from the skull and vertebra and then stored intact in 0.1 M PBS (pH 7.4). The position of the injury in the SC was measured from the caudal edge of the cerebellum to confirm that all animals were lesioned at the same level. A 2 cm segment was cut from the SC, with the lesion at the midpoint of this segment, and embedded in 1% (*w*/*v*) gelatin (Sigma-Aldrich). Using a CO2-freezing microtome (Polycut, Reichert-Jung, Australia), proximal and distal SC close to the grafts (1 cm) was cut sagitally in 40 *μ*m frozen sections, while the brain and brainstem were cut transversely in 50 *μ*m frozen sections. A consecutive series of sections were transferred to 24-well plates containing 0.1 M PB with 0.01% (*w*/*v*) sodium azide (Sigma-Aldrich) and stored at 4°C until processed for immunohistochemistry.

### 2.7. Tissue Analysis

#### 2.7.1. Axonal Transduction and Anterograde Tracing

Frozen coronal brain sections were examined for preinjury AAV-CNTF^mCherry^ or AAV-GFP transduction in the cortex and thalamus using immunofluorescence, in addition to post-SCI anterograde BDA labelling using both immunofluorescence and immunostaining with horseradish peroxidase (HRP). At the lesion site, longitudinal SC sections were similarly examined for axonal sprouting and regrowth (BDA). After blocking for 30 min in PBS containing 10% (*v*/*v*) normal goat serum and 0.02% (*v*/*v*) Triton X-100, AAV-transduced axons were immunolabelled overnight at 4°C using antibodies to mCherry (Living Colours, 1/600 in PBS). After washes, a secondary Cy3 goat anti-mouse antibody (Jackson ImmunoResearch 115613, 1/500 dilution in PBS) was applied for 30 min at room temperature, before unbound antibody was washed away and sections coverslipped. In most cases, BDA was visualized using commercial VECTASTAIN avidin-biotin (Vector Laboratories, USA, number PK-4000) kits (as per manufacturer's instructions) and horseradish peroxidase (HRP) histochemistry. Briefly, avidin-biotinylated (ABC)/HRP, followed by 3,3′-diaminobenzidine (DAB) solution was used to visualize BDA-stained axons. The ABC/HRP reagents were prepared 30 minutes before application to the sections. The sections were incubated with ABC reagents for 1.5 hours at room temperature and washed with PBS. A DAB solution (10% (*v*/*v*) DAB metal concentrate in peroxidase buffer) was then added to the sections and incubated for 5–15 minutes on a shaker. Sections were washed with PBS, allowed to air dry overnight and then counterstained with 1% (*w*/*v*) toluidine blue and coverslipped with DEPEX mounting medium (Fronine Lab Supply, Australia). To enable identification of both mCherry and BDA-labelled axons in the same longitudinal tissue sections, BDA was occasionally visualized using FITC-conjugated anti-streptavidin secondary antibodies (Thermo Fisher Scientific). In AAV-GFP-injected rats, brain and spinal cord sections were immunostained with an antibody to GFP (rabbit 1/500, Millipore, in PBS) followed by goat anti-rabbit IgG FITC-conjugated secondary antibodies (Jackson ImmunoResearch 89751, diluted 1/100 in PBS) similarly described as above.

In mCherry-, GFP-, and BDA-stained sections, the accumulation of debris and branching of CST axons were commonly seen rostral to the SCI and associated cysts. Axon sprouting was especially evident in AAV-CNTF-injected animals. In a series of sagittal sections, the rostral extent of this region was measured from the beginning of the injury site in 38 rats (*n* = 6 for AAV-GFP; *n* = 15 for AAV-CNTF^mCherry^; *n* = 6 for AAV-CNTF^mCherry^ + nonviable rMPCs; and *n* = 11 for AAV-CNTF^mCherry^ + viable rMPCs).

#### 2.7.2. Immunohistochemical Analyses of Glial and Neuronal Phenotypes

Brain and spinal cord tissue sections were blocked in 10% (*v*/*v*) fetal calf serum (Gibco, BRL) and 0.2% (*v*/*v*) Triton X-100 in PBS for 10 minutes at room temperature and washed in PBS. Primary antibodies (diluted at 1/500 in PBS unless otherwise stated) to confirm the expression of phenotypic markers for glial cells, GFAP in spinal cord (Millipore, AB3080), and axon populations using antibodies to *β*-III tubulin (Covance, PRB-435) in the brain and spinal cord were used. Detection using FITC- or Cy3-conjugated secondary goat anti-mouse or goat anti-rabbit antibodies (diluted at 1/400 in PBS, Jackson ImmunoResearch, 115613, 89751) as described previously [24, 25].

#### 2.7.3. Quantitative Analysis of Tissue Sparing in the Spinal Cord

At least two independent raters (blinded) also measured cyst sizes to remove bias. Tissue sparing was assessed by measuring cyst size and the amount of intact versus degenerating tissue [24, 25]. Briefly, assessment of spinal tissue sparing was carried out using 0.05% (*w*/*v*) toluidine and 0.005% (*w*/*v*) borax solution followed by dehydration in sequentially graded ethanol (*v*/*v*) of 70%, 90%, and 100%. Staining on every sixth sagittal section was used to determine the volume of spared spinal tissue. In each section, the total number of pixels in a 2.5 mm-long SC segment was determined, with the lesion epicenter in the middle, as well as the area of damaged spinal tissue around it. The border of the damaged tissue was defined by the absence of healthy cells and an obvious discontinuity in density. Measurements of each section were summed per rat and averaged to give the amount of spared tissue, and percentage was calculated as the difference between the area of damaged tissue versus the whole segment (field of view) [24, 25].

#### 2.7.4. Microscopy

A Nikon Eclipse E800 microscope was used to visualise immunofluorescence staining as well as confirm successful BDA injection into the brain and to visualize BDA-stained axons at the SCI site. The distances between the front edge of the most rostral cyst (if there was more than one) and any axons observed alongside or caudal to the cyst were measured using the NIS elements BR 4.5 software.

#### 2.7.5. Statistics

Using GraphPad Prism v4.03, SBSS (version 21.0) and InStat v3.06 for Windows (GraphPad Software, San Diego, USA), 1- and 2-way repeated measures using either one- or two-way analysis of variance (ANOVA) plus Tukey's post hoc analysis as required were performed, except for BBB scoring which uses Kruskal-Wallis analysis (nonparametric ANOVA) as described previously [24, 25]. In addition, Mann–Whitney post hoc testing was performed.

## 3. Results

### 3.1. Preinjury AAV Gene Therapy and Postinjury BDA Injection into the Cortex

**Figure 1:**
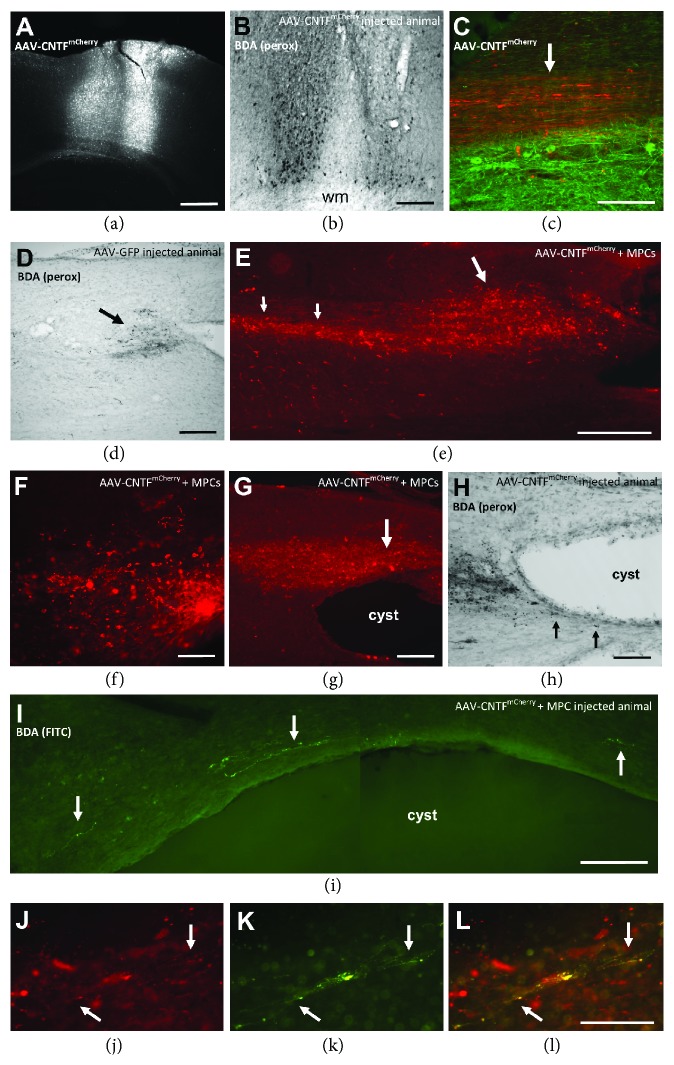
(a) Two AAV-CNTF^mCherry^ injections in the cerebral cortex. (b) BDA injections into the cortex revealed using immunoperoxidase; note the many labelled neurons in deeper layers. This animal had previously received cortical injections of AAV-CNTF^mCherry^. (c–l) Longitudinal sections of the spinal cord—in all cases rostral is to the left of the picture. (c) Section immunostained for mCherry (red) and *β*-III tubulin (green) showing anterogradely labelled mCherry-positive axons (arrow) in the dorsal corticospinal tract far rostral to the lesion site. (d) Control AAV-GFP-injected rat (no MPCs injected); a small number of immunoperoxidase BDA-labelled axons and debris are visible just in front of a rostral cyst (arrow), with no axons extending beyond the injury. (e–g) Large numbers of mCherry-positive axons rostral (e, f) and running dorsally over and beyond the cyst (g); these rats received AAV-CNTF^mCherry^ cortical injections plus an intraspinal injection of viable rat mesenchymal precursor cells (rMPCs). Note in (e) the profusion of mCherry-positive profiles (large arrow) approximately 1 mm rostral to the lesion cavity, growing into regions dorsal to the corticospinal tract (small arrows). There appears to be considerable sprouting of axons in this zone (f). (h) Immunoperoxidase BDA-labelled axons and debris rostral to a cyst in a rat injected with AAV-CNTF^mCherry^. Several axons can be seen running caudally, ventral to the cyst (arrows). (i) BDA-labelled cortical axons (arrows) visualized using a fluorescent secondary antibody (green), running over a cystic cavity. This animal also received AAV-CNTF^mCherry^ and viable MPC injections. (j–l) Cortical axons (arrows) double labelled (l) with both mCherry ((j), red) and BDA ((k), green); note, some axons are only mCherry or BDA immunoreactive. Scale bars: (a, e), 500 *μ*m; (b, h, and i), 200 *μ*m; (c, d, g, and j–l), 100 *μ*m; and (f) 50 *μ*m.

Both GFP and mCherry expression in transduced axons following AAV-GFP control and bi*cis*tronic AAV-CNTF^mCherry^ injections into the cortex prior to SCI were confirmed at day 56 post-SCI using immunofluorescence microscopy on relevant brain sections (Figure 1(a)). Note that the mCherry expression in neurons was confirmed through all the layers of cortex, including layer V containing CSN; this robust, post-2A linker expression is indicative of widespread transduction and expression of the secretable form of CNTF. BDA injection sites in the cortex were also visualized with ABC/HRP and DAB reaction (see Figure 1(b)) or immunofluorescence at day 56. BDA staining reached layer V of the cortex indicating the successful labelling of CSN and, as for mCherry expression, the label in the ventrolateral nucleus of the thalamus confirmed injection into appropriate sensorimotor cortical regions. The appearance of BDA in the same tracts in the cortex as transduced AAV-GFP control and AAV-CNTF^mCherry^ confirms that we were able to successfully label similar areas of axonal projection from the cortex that would facilitate identification within the CST regions of the spinal cord.

### 3.2. AAV-Transduced and BDA-Labelled Projections in the Spinal Cord

In all treatment groups, AAV-transduced, immunostained axons and BDA-immunolabelled axons were seen in the contralateral CST rostral to the injury site. An example is shown in Figure 1(c) with AAV-CNTF^mCherry^-labelled axons (red) in the CST adjacent to *β*III tubulin-positive fibers and neurons (green). While nearly all CST fibers were well aligned in the ventral dorsal column in segments far rostral to the SCI site (Figure 1(c)), immediately in front of the injury these axons became disorganized and more broadly distributed. In this region, in addition to degenerate axon profiles and other debris, apparently intact CST axons possessed complex, irregular profiles strongly suggestive of local sprouting and regenerative responses. In AAV-GFP control rats, there were small numbers of these axons immediately rostral to the first lesion cavity; however, in this group GFP-positive or BDA-labelled CST axons were *never* seen caudal to the injury/cyst. Furthermore, compared to animals injected with AAV-CNTF^mCherry^ (Figures 1(e)–1(g)), there was greater axon dieback as well as relatively little sprouting. The best example is shown in Figure 1(f) (BDA label). By comparison, in rats with AAV-CNTF^mCherry^ cortical injections, there was consistently a much higher density of CST axons 400–1000 *μ*m rostral to the first spinal cord cavity, as revealed in both mCherry- (Figures 1(e)–1(g)) and BDA- (Figure 1(h)) immunostained material.

In a series of mCherry- or BDA-immunostained sagittal sections, the rostral extent of the zone containing scattered, often branched, CST axons was measured from the beginning of the injury site (taken as the rostral edge of the first lesion cavity) in 38 rats. These “expanded” zones of CST label (Figures 1(e)–1(h)) were seen in 3/6 AAV-GFP-injected rats, 9/15 AAV-CNTF^mCherry^ rats, 4/6 rats with AAV-CNTF^mCherry^ + nonviable rMPCs, and 8/11 rats with AAV-CNTF^mCherry^ + viable rMPCs. The mean rostral extent of this zone was 323 ± 125 *μ*m (S.D.), 483 ± 226 *μ*m, 475 ± 330 *μ*m, and 638 ± 370 *μ*m for the four groups, respectively. There was considerable interanimal variability as shown by the large standard deviations, nonetheless the trend for increased density and greater areal extent of rostral CST sprouting in AAV-CNTF-injected animals is evident.

After AAV-CNTF but not AAV-GFP cortical injections, many labelled CST axons were located beyond the rostral edge of the cyst (Figure 1(g)). Growth of BDA-positive axons beyond the cyst was also seen (arrows, Figure 1(h)). Long-distance growth of axons was also occasionally seen as shown in Figure 1(j); again, note the irregular nature of the postinjury BDA^FITC^-labelled axonal profiles. This animal also received a viable MHC graft. In sections immunostained for both mCherry (red) and BDA (FITC—green), we observed occasional axons that were both AAV-CNTF^mCherry^- and anti-BDA^FITC^-positive (arrows, Figures 1(j)–1(l)). In the example shown, the axons were located ventral to a cyst. The double labelling indicates that at last some of the mCherry and BDA cortical injections were successfully made in overlapping regions of cortex, resulting in the dual label of the projecting layer V pyramidal neurons.

**Figure 2:**
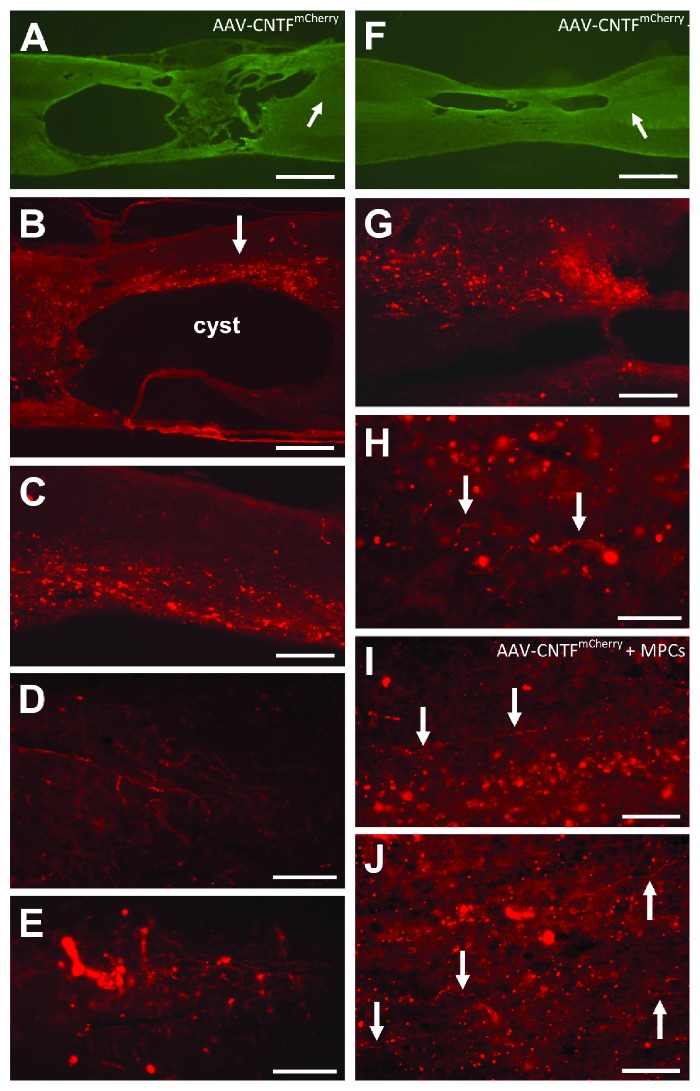
Two examples (A–E and F–H) of animals that received cortical AAV-CNTF^mCherry^ injections and with mCherry-positive corticospinal axons distal to spinal cysts (D, E, I and J), and therefore distal to the initial injury. In all images rostral is to the left. (A, F) Low power views of cysts (*β*-III tubulin-immunostained sections) in each rat; the arrows in (A) and (B) point to the approximate location of the axons shown in (D, E, and H), respectively. (B, C) Large numbers of mCherry-positive axons (arrow) dorsal to the large rostral cyst (see (A)), with small numbers of irregularly organized axons distal (D, E). (G) mCherry-labelled axons rostral and dorsal to the cyst, with several axons (arrows) located distal to the injury (H). In one animal injected with AAV-CNTF^mCherry^ and that received a spinal rMPC injection, numerous mCherry-positive axons (arrowed) were seen distal to the most caudal lesion cavity (I, J). Scale bars: (A, F), 1 mm; (B), 500 *μ*m; (C, G), 200 *μ*m; (D, E, I, and J), 100 *μ*m; and (H) = 50 *μ*m.

Figure 2 shows representative longitudinal spinal cord sections from three animals treated either with AAV-CNTF^mCherry^ alone (Figures 2(A)–2(H)) or AAV-CNTF^mCherry^ + viable rMPC transplantation (Figures 2(I), and 2(J)). Low power images show the size and location of cysts in two of these animals (Figures 2(A) and 2(F), resp.). The arrows in Figures 2(A) and 2(F) point to the approximate postcyst location of the mCherry-positive axons shown in Figures 2(D), 2(E), and 2(I). In the rat shown in Figures 2(A)–2(E), there were large numbers of mCherry-positive fibers dorsal to the first large cyst in regions of spared tissue midway within the lesion site (arrow, Figure 2(B), shown in higher power in Figure 2(C). More importantly, we also observed AAV-CNTF^mCherry^-positive fibers in the spinal cord distal to the lesion (Figures 2(D) and 2(E)). These fibers were nonlinear in orientation and, although infrequent, they were located up to 3-4 mm beyond the rostral edge of the injury site. In the animal shown in Figures 2(F)–2(H), again there were many mCherry-immunopositive axons located just in front of, and dorsal to, the rostral cavity (Figure 2(G)), and many sprouting BDA-positive axons were also seen. However, in this animal, there were only a small number of mCherry-labelled axons located caudal to the injury site (arrows, Figure 2(H)). It is worth emphasizing in Figures 2(D), 2(E), and 2(I) that the irregular course and branching of these axons are strongly suggestive of regenerative growth. One other rat, this one treated with both AAV-CNTF^mCherry^ and viable rMPCs, possessed a number of mCherry-labelled axons distal to the contusion injury and to the most caudal lesion cavity (arrows, Figures 2(I) and 2(J)).

### 3.3. Functional Hindlimb Recovery Is Generally Enhanced after CNTF Gene Therapy

#### 3.3.1. Ladderwalk

**Figure 3:**
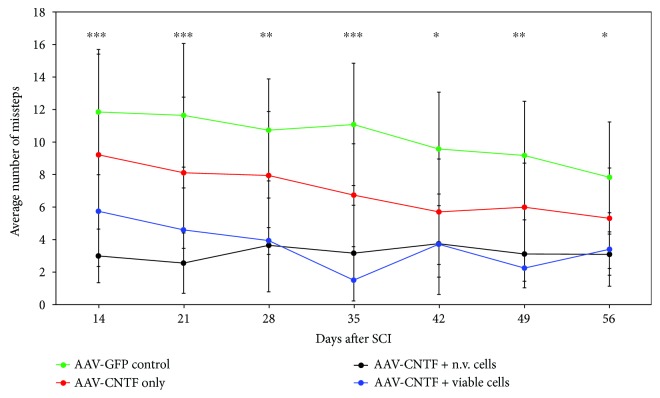
Functional hindlimb recovery promoted after AAV-CNTF gene therapy and cellular transplantation as assessed by Ladderwalk: reduced misstepping over the Ladderwalk apparatus indicates that AAV-CNTF^mCherry^ therapy (red, crosses) promoted significant functional improvement after SCI compared with control AAV-GFP treatment (green). Note that at day 14, the Ladderwalk testing was carried out prior to injection of either viable (blue) or nonviable (black) cells. Post hoc tests revealed statistically significant differences between the AAV-GFP-treated control group and AAV-CNTF^mCherry^ + nonviable rMPCs at all time points as well as between the AAV-GFP-treated control group and AAV-CNTF^mCherry^ + viable rMPCs at all time points except day 56 (^∗^*p* = 0.01–0.05, ^∗∗^*p* = 0.005–0.01, and ^∗∗∗^*p* = 0.001–0.005). Two-way repeated measures ANOVA was conducted using PRISM (time: *p* = 0.0001, treatment: *p* = 0.0016, and interaction: *p* = 0.0001). Standard deviation is shown.

The number of missteps over the Ladderwalk apparatus showed a gradual decline for all treatment groups from day 14 through to endpoint at day 56 after SCI (Figure 3). Animals treated with control AAV-GFP after SCI had the highest misstep counts across all time points, with the average decreasing gradually from 12 at day14, to 8 at day 56, respectively. Rats with SCI followed by AAV-CNTF^mCherry^ treatment consistently displayed a lower average number of missteps than control AAV-GFP treatment, decreasing gradually from ~9 at day14 to ~6 at day 56, respectively. The combined AAV-CNTF^mCherry^ treatment with either nonviable or viable rMPC transplantation at the lesion site markedly reduced the average numbers of missteps, ranging from 3 to 6 at day 14 after SCI, with both of these groups showing very similar average missteps of 3 from day 28 until day 56 after SCI. Two-way ANOVA showed statistically significant differences (pairwise comparisons) between all groups from day 14 until day 56 after SCI (*p* = 0.002, 0.003, 0.006, 0.001, 0.013, 0.002, and 0.032 at days 14, 21, 28, 35, 42, 49, and 56, resp.). Post hoc tests revealed statistically significant differences between the AAV-GFP-treated control group and AAV-CNTF^mCherry^ + nonviable rMPCs at all time points as well as between the AAV-GFP-treated control group and AAV-CNTF^mCherry^ + viable rMPCs at all time points except day 14 and day 56 (Figure 3).

Because the two-way ANOVA with repeated measures described above showed a significant interaction (*p* = 0.0001), we then separated the factors and performed a one-way ANOVA on all AAV-CNTF^mCherry^-treated animals at day 14 only. This one-way ANOVA revealed that there was no difference between the cell-type (no cell, viable, and nonviable rMPC) treatments for the AAV-CNTF^mCherry^-treated cohort at day 14 (*p* = 0.395), to be expected given that the Ladderwalk tests were performed prior to rMPC transplantation at this time point. Note that the number of animals per group at day 14 was less than at later times because only those animals that supported their weight on their hindlimbs could be tested. Additionally, the one-way repeated measures ANOVA within the AAV-CNTF^mCherry^ animals revealed that while there was an overall effect of a reduction in missteps over time (*p* = 0.001), there was no difference between the cell-type treatment groups (*p* = 0.143). Interestingly however, in the AAV-CNTF^mCherry^-injected rats only the AAV-CNTF^mCherry^ without rMPC group showed a significant reduction in missteps (*p* = 0.05, LSD test) across time with post hoc testing.

#### 3.3.2. Open-Field Locomotion (BBB)

**Figure 4:**
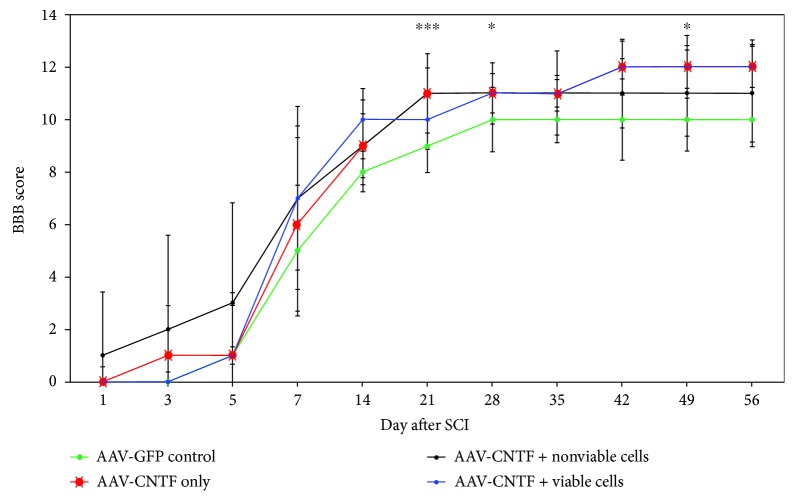
Functional hindlimb recovery promoted after AAV-CNTF gene therapy and cellular transplantation as assessed by open-field locomotion (BBB). Significant functional improvements were observed following AAV-CNTF^mCherry^ (red), AAV-CNTF^mCherry^ + nonviable rMPC (black), and AAV-CNTF^mCherry^ + viable rMPC (blue) treatment compared to AAV-GFP-treated control animals (green) generally from day 21 after SCI onwards, although statistically significant differences were not maintained at all time points (^∗^*p* = 0.01–0.05 and ^∗∗∗^*p* = 0.001–0.005). Standard deviation is shown.

BBB scores for hindlimb recovery (Figure 4) revealed significant differences between the AAV-GFP-treated control group (green) compared to the AAV-CNTF^mCherry^ (red, crosses), AAV-CNTF^mCherry^ + nonviable rMPC (black), and AAV-CNTF^mCherry^ + viable rMPC (blue) treatment groups, generally from day 21 onwards. All treatment groups followed a slow increase in average BBB scores (0-1 at day 0 following SCI) until around day 5–day 7 when average scores began to typically increase at a greater rate, with the AAV-GFP-treated control group consistently showing the lowest average BBB scores of 5 from day 7 (which corresponds to a slight movement of two joints and an extensive movement of the third joint) until score 10 at day 56 after SCI (which corresponds to plantar stepping with occasional weight bearing and no forelimb-hindlimb coordination). AAV-CNTF^mCherry^ and AAV-CNTF^mCherry^ + nonviable rMPC groups' scores plateaued from day 21 after SCI with an average BBB score of 11 (which corresponds to plantar stepping with frequent to consistent weight bearing and NO forelimb-hindlimb coordination). Only the AAV-CNTF^mCherry^ and AAV-CNTF^mCherry^ + viable rMPC treatment group obtained higher scores from day 42 until day 56 after SCI, where average BBB scores plateaued at 12 (which corresponds to plantar stepping with frequent to consistent weight bearing and occasional forelimb-hindlimb coordination), suggesting that CNTF gene therapy alone and CNTF gene therapy + viable MPC transplantation into the lesion resulted in the best functional outcomes. Kruskal Wallis scores showed overall statistically significant differences between the AAV-GFP-treated control group and all other treatment groups at day 21 (^∗∗^*p* = 0.006), day 28 (^∗∗∗^*p* = 0.0016), and day 49 (^∗^*p* = 0.023). Specific Mann–Whitney post hoc tests revealed statistically significant differences between the AAV-GFP-treated control group and AAV-CNTF^mCherry^ (*p* = 0.001 at day 21, *p* = 0.004 at day 28, and *p* = 0.003 at day 49), AAV-CNTF^mCherry^ + nonviable rMPCs (*p* = 0.018 at day 21, *p* = 0.036 at day 28, and *p* = 0.113 at day 49), and AAV-CNTF^mCherry^ + viable rMPCs (*p* = 0.021 at day 21, *p* = 0.008 at day 28, and *p* = 0.059 at day 49), respectively.

#### 3.3.3. Ratwalk® Gait Analysis

**Figure 5:**
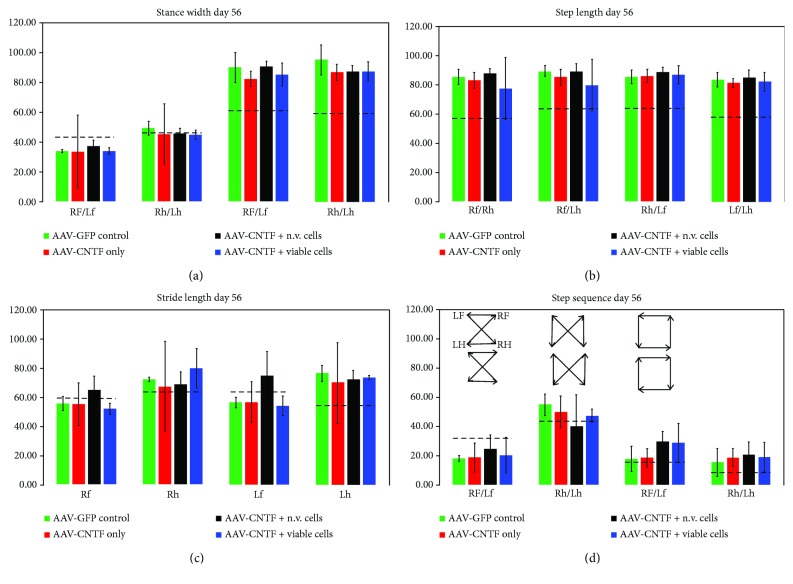
Ratwalk gait analysis at day 56 after SCI. Averages of arbitrary unit values of each treatment group compared to preinjury levels (dotted black lines) are shown for stance width (a), step length (b), stride length (c), and step sequence (d). Despite no statistically significant differences *between* any treatment group for any variable, compensatory changes such as a marked reduction in the stance width (a) of the forelimbs (Rf/Lf) between opposing fore- and hindlimbs (Rf/Lh and Lf/Rh) in all groups compared to preinjury levels were supported by data for step length (b) and stride length (c). A general decrease in the amount of patterns of coordinated fore- and hindlimb placement (“cruciate,” “alternate,” and “rotary” as indicated in (d)) and an increase in the amount of noncoordinated fore- and hindlimb placement (“none”) was observed. Standard deviation is shown.

Ratwalk analysis on animals showing hindlimb weight support are summarized in Figure 5, with averages of arbitrary unit values assigned by the software *in silica* for each treatment group compared to preinjury levels (dotted black lines) shown for stance width (5A), step length (5B), stride length (5C), and step sequence (5D) at day 56 after injury, by which time any differences in gait parameters should be apparent. Stance width is the average distance between each of the forelimbs (Rf/Lf), each of the hindlimbs (Rh/Lh), and each of the fore-to-hindlimb placements (Rf/Lh and Lf/Rh). There was no significant difference between any treatment group for any variable in stance width at day 56. Typically, hindlimb stance width increases slightly very early on after SCI (data not shown) and although the AAV-GFP-treated control group still showed a marginally increased average hindlimb stance width compared to all other treatment groups, there was no statistical difference between them or preinjury levels. A higher average stance width between opposing fore- and hindlimbs (Rf/Lh and Lf/Rh) was maintained at day 56 post-SCI for all treatment groups compared to preinjury levels and suggests that all animals assume a longer distance between each fore- and hindlimbs as a consequence of the injury irrespective of treatment. The stance width data suggesting these compensatory fore- and hindlimb changes in the treatment groups is confirmed by the day 56 post-SCI analyses for step length (Figure 5(b)) and stride length (Figure 5(c)), which again showed no statistically significant differences between treatments. Step length analysis shows a higher average distance between Rf/Rh, Rf/Lh, Rh/Lf, and Lf/Lh across all groups compared to preinjury levels with no statistically significant differences between the groups (Figure 5(b)). Stride length also shows that generally forelimb strides are shorter and hindlimb strides are longer on average compared to preinjury levels (Figure 5(c)), consistent with the idea that compensatory movements to bring forelimbs closer and take shorter strides to help “pull” the animal forward correlates with longer hindlimb strides that adopt a wider stance (for more stability) and therefore longer distances. While the AAV-CNTF^mCherry^ + nonviable rMPC group of animals still showed slightly higher forelimb distances compared to preinjury levels, there were no statistically significant differences between treatments.

Step sequence analysis revealed that following SCI, there was generally a decrease in the amount of patterns of coordinated fore- and hindlimb placements designated as “cruciate,” “alternate,” and “rotary” using the Ratwalk software, as well as an increase in the amount of unrecognisable (noncoordinated) fore- and hindlimb placements designated as “none” (Figure 5(d)). There remained a significant decrease in the amount of cruciate patterns of sequence compared to preinjury levels, and despite some variation between treatment groups, there were no statistically significant differences between treatment groups for both alternate and rotary patterns of step sequence. The amount of uncoordinated patterns of step sequence (“none”) was almost twice as high for all treatment groups at day 56 post-SCI, again with no statistically significant differences between treatment groups.

### 3.4. Transplantation of Viable rMPCs, but Not Cortical Gene Therapy, Promotes Tissue Sparing

**Figure 6:**
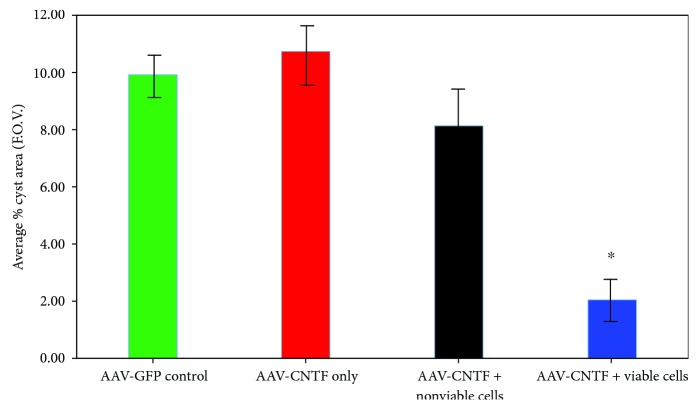
Tissue sparing promoted by combined cortical AAV-CNTF^mCherry^ therapy and local transplantation of viable rMPCs. AAV-GFP control treatment and AAV-CNTF^mCherry^ treatment groups revealed similar average areas of cyst formation in spinal cord sections stained by toluidine blue, and although AAV-CNTF^mCherry^ + nonviable rMPC treatment resulted in slightly lower average cyst sizes, only AAV-CNTF^mCherry^ + viable rMPC treatment into the lesion resulted in a statistically significant reduction in average cyst size. ^∗^*p* = 0.01–0.05.

Cysts developed at the injury site of all animals in all groups after SCI. The cyst sizes measured at day 56 after SCI are shown in Figure 6. AAV-GFP treatment and AAV-CNTF^mCherry^ treatment groups on average had a 10-11% area of field of view occupied by cysts, respectively. While AAV-CNTF^mCherry^ + nonviable rMPC-treated animals still showed slightly lower average cyst sizes of about 8%, there was no statistical difference between any of these groups. However, AAV-CNTF^mCherry^ transduction in the cortex followed by *viable* rMPC transplantation into the lesion did result in a statistically significant reduction in average cyst size, to just a 2% area of field of view, indicating that viable rMPCs are able to significantly alter the terrain of the lesion site to affect tissue sparing. Note that two of the three rats with mCherry-positive axons distal to the injury site were in the AAV-CNTF^mCherry^ + viable rMPC group. Donor rMPCs were not labelled with any marker for identification posttransplantation, and there was no evidence of individual donor rMPCs remaining in spinal cord sections subjected to immunohistochemistry or toluidine blue staining (data not shown).

## 4. Discussion

In this study, we used AAV-CNTF^mCherry^ therapy to transduce neurons, including CSN, in the sensorimotor cortex of animals with a moderate thoracic (T10) contusion injury, with the aim of enhancing plasticity and promoting the regrowth of corticospinal tract axons after SCI. It is widely acknowledged that combinations of therapies are required for effective treatment of SCI, thus in some animals AAV-CNTF^mCherry^ was applied in combination with viable or nonviable rat mesenchymal precursor cells (rMPCs) grafted into the spinal lesion site two weeks after SCI to modulate the local inhibitory environment. As discussed more fully below, different types of AAV vectors have previously been injected into the cortex in SCI studies, and MPC grafts have also been tested after SCI, but to our knowledge no trials have—until now—combined these therapeutic approaches. AAV-CNTF^mCherry^ therapy alone or in combination with either viable or nonviable rMPC transplantation provided a sustained improvement in functional outcome over AAV-GFP alone as measured by Ladderwalk. Note that with this behavioural test, while control and CNTF treatments differed, there was no significant difference between the different AAV-CNTF^mCherry^ groups (no cells, viable cells, and nonviable cells), thus it seems that the presence of cortical CNTF was sufficient to yield an improvement in the stepping function. AAV-CNTF^mCherry^ therapy alone and in combination with rMPC transplantation also yielded sustained improvements as assessed by open-field locomotion (BBB). Ratwalk gait analyses revealed subtle compensatory mechanisms for limb placement after injury, but it is likely that even the moderate injury used here was too severe to reveal significant statistical differences. The average cyst size was significantly reduced only in the AAV-CNTF^mCherry^ + viable rMPC group.

In addition to many degenerative profiles, the sprouting of apparently intact axons was evident in the CST immediately rostral to the injury site. Compared to AAV-GFP controls, considerably more BDA or mCherry-labelled axons with complex profiles were located just rostral to the cysts in the AAV-CNTF^mCherry^-injected rats containing CNTF-transduced cortical neurons. Not only was the density of these axons higher, but the area of the spinal cord containing them extended further rostrally into uninjured segments. Furthermore, only in AAV-CNTF^mCherry^-transduced treatment groups were BDA and mCherry-immunoreactive axons seen to project alongside, and sometimes several millimeters beyond, the rostral border of the lesion-induced cysts. It is important to emphasize that, compared to oriented and organized fibers in the CST far rostral to the injury site, axons were more irregularly organized immediately in front of, as well as caudal to, cysts, strongly suggestive of local sprouting and regenerative responses.

In three of the eleven animals that received cortical AAV-CNTF^mCherry^ injections, axons were seen caudal to all cysts, located up to 3-4 mm beyond the rostral edge of the injury site. These axons were seen in two non-rMPC grafted animals as well as in a rat that also received an rMPC transplant. We did not detect such axons in AAV-GFP-injected animals, again supporting the suggestion that CNTF has the potential to enhance axon regeneration in the spinal cord by transducing appropriate neuronal populations in the sensorimotor cortex. In other CNS systems, vector-mediated delivery of CNTF to parent cell bodies combined with the transplantation of an appropriate bridging substrate (peripheral nerve), promotes the long-distance regeneration of injured axons [7–10], with at least some functional outcomes [11]. Note in particular that the survival effects of CNTF have been demonstrated on numerous neuron populations [14–16], including increasing the viability of corticospinal neurons in the sensorimotor cortex after intracortical axotomy [6]. Because AAV-CNTF delivery is only to the cortex, the primary site of trophic action is at the cell bodies, and transport and release of CNTF at axon tips likely occurs at a low level (we have confirmed this previously, data not shown). Based on previous studies in the visual system, we propose that the mechanisms of protection and promotion of plasticity and regenerative capacity are mediated and amplified both by direct infection of CSN themselves but also due to bystander release of CNTF by adjacent nonprojecting cortical cells [8, 39]. Many of the biological actions of CNTF are signalled via STAT3, and it is therefore important to note that vector-mediated delivery of a hyperactivated STAT3 enhances process outgrowth in cultured cortical neurons [40], and in vivo overexpression of STAT3 enhances CSN plasticity in mice after SCI [41].

Delivery of AAV vectors to the cortex has been reported by a number of groups, some to test the optimal serotype for transduction of cortical neurons, including CSN [42, 43], others to deliver factors designed to enhance CST repair after SCI [44–49]. Such studies are nonetheless much less frequent than those involving the delivery of vectors and/or growth factors to the spinal cord injury site itself [2, 50]. In some initial studies, we tested the effect of the cortical injection of AAV1 expressing insulin-like growth factor 1 (IGF-1) on CST plasticity after SCI, but although we saw some additional sprouting rostral to the injury, this was less than that seen after the cortical delivery of AAV-CNTF vectors, and we saw no significant changes in functional recovery (unpublished data). Interestingly then, postlesion AAV-assisted coexpression of IGF1 and osteopontin in cortical neurons resulted in robust CST regrowth and the recovery of CST-dependent behavioural outcomes after SCI [50], again indicative of the therapeutic power of combined therapies.

The long-term transduction of murine cortical neurons using an AAV vector to suppress or conditionally delete expression of phosphatase and tensin homolog (PTEN), the main suppressor of the PI3K-Akt survival and growth pathway, leads to the greater regenerative growth of CST axons and some functional improvements [45]. However the resultant long-term upregulation of protein kinases such as mTOR, a key regulator of protein translation in neurons, leads to some aberrant growth [48] and progressive changes in the growth of cells, their dendrites, and their axons [46]. This is not dissimilar to the effect of long-term (up to one year) AAV-mediated expression of CNTF on retinal ganglion cells, where there are not only changes in the dendritic morphology of transduced and neighboring nontransduced cells [51], but there is also altered expression of endogenous retinal genes [52]. Whether such effects are also seen in cortical neurons transduced with AAV-CNTF is yet to be determined, but this is an important topic for future studies.

MPCs from the bone marrow stroma have the potential to differentiate into cells with many of the phenotypic characteristics of neural tissue [53–55], migrate and integrate into CNS tissues, and express markers typical of mature neurons and astrocytes [56]. MPCs have been successfully transplanted into the spinal cord and shown to (a) promote regeneration of lesioned axons into the graft, (b) differentiate into neurons, (c) remyelinate damaged myelin sheaths around CNS axons, and (d) improve functional outcomes after SCI (for extensive reviews, see [57, 58]). Our own work using purified (Stro-1+) human MPCs from the bone marrow stroma of SCI patients dramatically improved anatomical (characterized by smaller cyst sizes, as well as lower amounts of degenerative tissue) and functional recovery after both acute and subacute/chronic SCI in nude rat (T-cell immunodeficient) hosts after contusion SCI [24, 25]. Rat MPCs can be prepared to an essentially pure, minor subpopulation of adult cells [59–61] significantly lessening any potential variation in functional and morphological outcomes, often encountered in the literature when using different hMPC donor cells in transplantation experiments.

rMPC transplantation at day 14 post-SCI presumably avoids the cytotoxicity of the acute injury and presumably allows sufficient time for targeted expression of CNTF to be switched on in transduced cortical cells [2, 4, 28]. Generally, SCI studies show significant results attributable to transplantation within a few weeks of injection [24, 25, 57, 62, 63]; however, in the present study the impact of rMPCs grafted into the injury site was evident in reducing postlesion cavity dimensions, but effects were absent or inconsistent in the behavioural studies involving AAV-CNTF^mCherry^-injected animals. It should be noted that a caveat of this study is that behavioural tests were continued after BDA was injected which could potentially obscure or increase treatment effects due to the injection-associated injury and surgery, although we did not observe any apparent reduction in functional outcomes between BDA injection and the final time points analysed. There did not appear to be greater macrophage/microglial reactivity associated with AAV transduction (either AAV-GFP-treated control or AAV-CNTF^mCherry^ treatments) beyond that typically observed in our other SCI studies [24, 25]. MPCs do produce some CNTF [64] but our experience is that, while MPCs alter the local environment to enhance axonal regrowth, few survive in the long term [24, 25]. Only viable rMPC grafts promoted statistically significant tissue sparing, yet nonviable rMPC transplantation had similar effects as viable rMPC transplantation in functional outcomes (as shown in our Ladderwalk and open-field assays). The use of (freeze-thawed) nonviable cells as appropriate controls for cell transplantation studies in SCI is by no means common. There is evidence to suggest that even fibroblasts can contribute to functional and/or morphological improvements in animal models of SCI compared to specific stem/precursor cell types [57, 63]. Indeed some studies have reported functional improvements without associated (and expected?) structural improvements, and vice versa [57, 65]. A possible scenario is that MPCs (which are known to have immunosuppressive properties [66]) may act as immune “decoys” that modulate the host immune response (e.g., [67]) and this property may still be effective even if the cells themselves are not viable. This could potentially allow the “normal” host repair mechanism to be more effective and be reflected in either functional and/or morphological outcomes.

### 4.1. Impact and Future Direction

The combination of AAV-targeted expression of CNTF, with or without the use of stem cell graft technology, represents a novel strategy to assess the effect of vector-mediated production of growth factors on plasticity and regeneration after SCI. A major aim of these experiments was to assess the capability of cortical gene therapy to promote potential plasticity and regrowth/regeneration of CST axons. The present behavioural and morphological data after thoracic contusion injury show the promise of using cortical AAV-CNTF gene therapy to promote repair after SCI. In future studies, we will test this approach in a model of cervical CST hemitransection, focusing on the importance of this tract in rodent forelimb function. The data presented here aid in the advancement of technologies related to the development of more effective gene therapy and begin to provide a platform for exploring the possibility of preclinical studies aimed at using gene therapy to modify cortical neurons as part of an SCI repair strategy.

## Data Availability

The histological slides are stored in Stuart I. Hodgetts' laboratory, as are videos of behavioural analyses.
